# Guideline developers are not the only experts: Utilising the FRAM method in sepsis pathways

**DOI:** 10.1186/s12916-018-1212-6

**Published:** 2018-11-21

**Authors:** Damian Roland

**Affiliations:** 1 SAPPHIRE, Health Sciences, University of Leicester, Leicester, LE1 7RH UK; 2 Paediatric Emergency Medicine Leicester Academic (PEMLA) Group, Children’s Emergency Department, Leicester Royal Infirmary, Infirmary Square, Leicester, LE1 5WW UK

**Keywords:** Sepsis, Complexity, FRAM, Clinical outcomes

## Abstract

Improving clinical outcomes and quality of care in diseases such as sepsis, which are heterogeneous in their presenting signs and symptoms, is a challenge. One approach is to utilise the Functional Analysis Resonance Method (FRAM), which enables systems to examine process and sociocontextual issues in treatment and management pathways. McNab et al. applied FRAM to group of primary care (family) practices to understand the barriers and enablers in the management of sepsis and determined a suite of possible interventions that might improve patient care. This commentary reviews the FRAM process and highlights some core issues for systems to consider when tackling diseases like sepsis.

Please see related article: https://bmcmedicine.biomedcentral.com/articles/10.1186/s12916-018-1164-x.

## Background

**Fig. 1:**
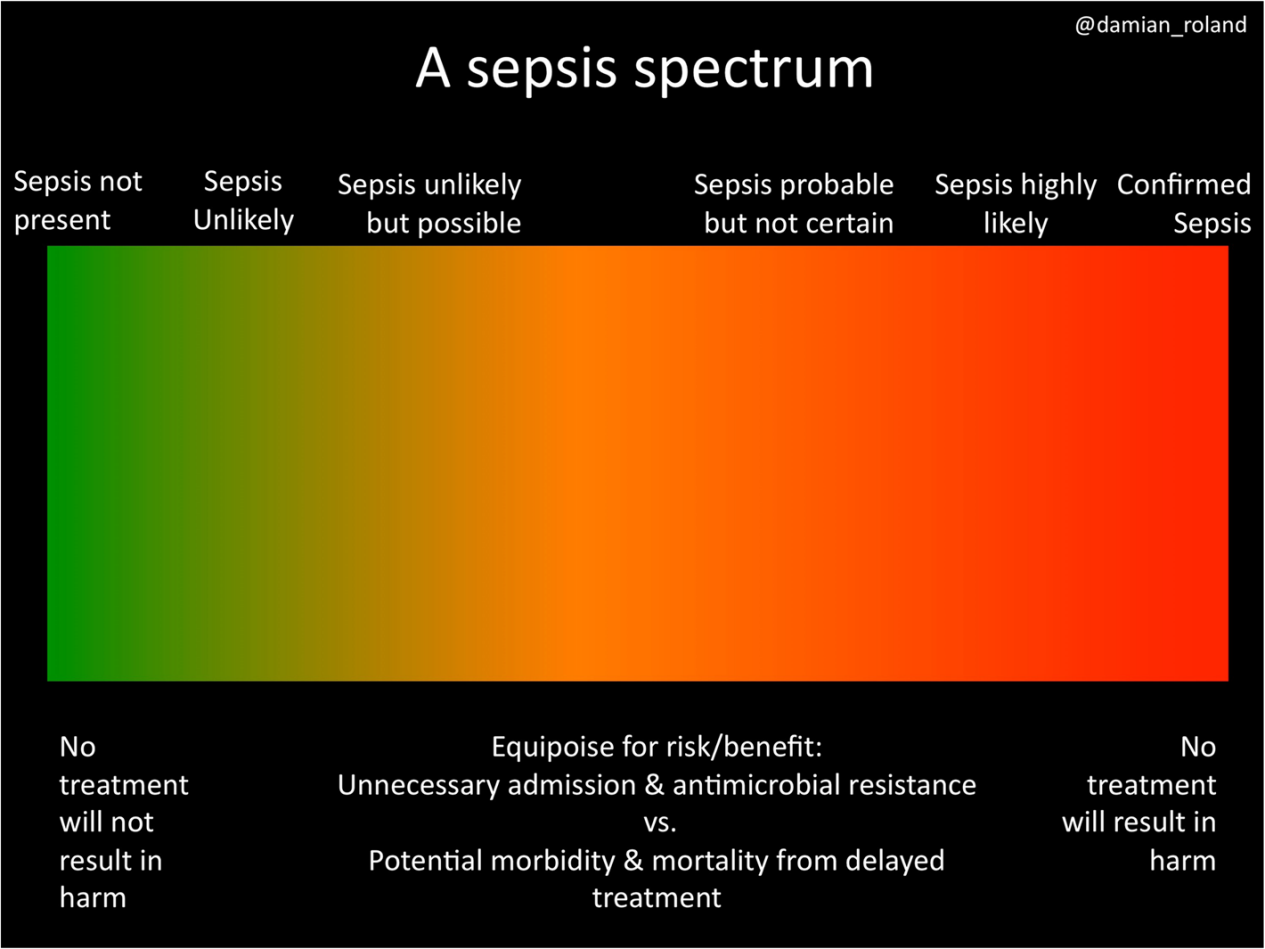
The sepsis spectrum, an original concept by Dr Damian Roland [3]

Sepsis may be defined as life-threatening organ dysfunction caused by a dysregulated host response to infection [1]. Improving the care of patients with sepsis is challenging. The continuing debate over its definition [2] means sepsis remains a diagnosis of retrospect because the initial presenting features are often consistent with a variety of diagnoses. This has created significant challenges in deriving systems that are specific enough to not overload services with patients who do not require treatment (or, even worse, who have a different but equally significant diagnosis), but sensitive enough to maintain patient safety. The increased profile of sepsis has resulted in a demand for practice change from regulators and health services. This change is welcome because the mortality and morbidity burden of the disease, in particular in adult practice, is high. However, strategies to improve care have not always followed improvement science principles; that is, solutions have been implemented with little understanding of the context of the environment in which care is delivered. Too often little attention is paid to processes which may inhibit clinicians’ ability to make accurate judgements. Furthermore, sepsis is not always a binary condition; there is a spectrum of risk that clinicians must consider in their decision making (Fig. 1 [3]).

The study by McNab and colleagues [4] is therefore very welcome because they examine an underexplored area of sepsis recognition (primary care). They have also used an approach that is likely to reduce unsuitable and perhaps counterproductive knee-jerk interventions.

The Functional Analysis Resonance Method (FRAM) facilitates awareness of the complexity of systems and is especially useful at driving focused improvement to a problem. Essentially, it is a variety of mixed-methods approaches that may include semi-structured interviews, focus groups and ethnographic methods to identify all possible functions of a system and their relevant interactions. This produces a model of how complex system interdependencies align and how workflows influence each other [5, 6].

### FRAM score and team behaviour

McNab et al. predicted that any involvement of an Early Warning Score (EWS) was likely to fail, unless it could afford the users flexibility in the interpretation of the scores. The study identified that a mandated score led to a projected pathway that was often dissonant to the clinician’s own beliefs about patient’s acuity. This dissonance led to behaviours that avoided applying the correct use or interpretation of the EWS. FRAM is also useful to highlight dynamics within teams that the team members may not be sighted to without external review. For example, in one of the primary care practices, clinical personnel trusted their administrative staff to be able to recognise the most unwell patients and direct them to appropriate care facilities. However, the administrative staff felt they could not do this and were not trained to do so. They may have possessed proficient skills to undertake some form of triage, but did not act as a triage system in the way supposed by their clinical colleagues.

### ‘Work as imagined’ versus ‘work as done’

The study by McNab et al. highlights a particular issue that plagues health policy decision making: the lack of awareness of the difference between ‘work as done’ and ‘work as imagined’. Within all healthcare settings, external parties hold beliefs about the patient care process. While well intentioned, these are not always based on a realistic view of the system in question. To maintain efficiencies in their service, members of clinical and administrative staff may enact workarounds. These may include avoiding basic tasks perceived to have low value, skipping middle steps in guidelines or escalating care through non-standard processes. Because primary care physicians are generally experienced enough to know whether or not their patients can be safely sent home, some primary care patients may not have a full set of vital signs taken. Appropriately some patients (with unrecorded abnormal vital signs) are not investigated or referred to secondary services because they do not have sepsis and will come to no harm. Mandating a set of observations for all patients will reveal those patients with a documented physiological abnormality (meaning they require further investigation as per sepsis protocols) but who previously would have been safely sent home.

“*But people want every box ticked. Because someone will audit it, someone will look at it and then they will come round and go – like we had a complaint from a patient who had a sore throat… turned out, two days later, he had quinsy, but you don't seem to have recorded saturations on him*.” –– Participant in the McNab et al. study [4].

### Contribution of the FRAM Process

The FRAM process can also elucidate why these workarounds can be harmful as well as having benefits. While the implementation of guidance, such as taking mandated, ubiquitously applied vital signs, can lead to unnecessary over-referral, this physiological information is important to admitting teams (something that might not be immediately apparent to the referrer). By the very nature of the disease process, some patients may indeed have sepsis despite being thought well by the primary care clinician. On these occasions, taking a set of vital signs may prompt an intervention that was not previously considered. This balance (over-referral versus individual patient risk) may be self-evident, but its exploration through the FRAM process can highlight the relevant problems to all parties. Shared, equitable and balanced solutions can then be implemented.

## Conclusions

For sepsis, FRAM highlighted the potentially unrealised gap between ‘work as imagined’ and ‘work as done’. However, there are other disease processes or presenting conditions (such as chest pain) where the implementation of evidence-based pathways must appreciate the underlying system process of a given clinical environment. What FRAM really highlights is who the experts are: they are not just the developers of guidance, they are also the members of staff doing the work and the patients being treated. The experiences of both staff and patients will develop and facilitate the changes likely to be most beneficial to all parties.
